# Insights into Evolutionary, Genomic, and Biogeographic Characterizations of Chryseobacterium nepalense Represented by a Polyvinyl Alcohol-Degrading Bacterium, AC3

**DOI:** 10.1128/spectrum.02179-22

**Published:** 2022-08-24

**Authors:** Xinbei Liu, Dandan Wang, Zhiqiu Yin, Li Sun, Shiqi Pang, Jianing Liu, Wei Li, Shiyu Cui, Weiwei Huang, Yuhui Du, Zhihong Xie

**Affiliations:** a National Engineering Research Center for Efficient Utilization of Soil and Fertilizer Resources, College of Resources and Environment of Shandong Agricultural University, Tai’an, China; b College of Plant Protection, Shanxi Agricultural University, Taiyuan, People’s Republic of China; c School of Biology and Biological Engineering, South China University of Technology, Guangzhou, China; Dublin City University

**Keywords:** biodegradation, biogeographic distribution, comparative genomics, environmental adaptation, *Chryseobacterium*, polyvinyl alcohol

## Abstract

*Chryseobacterium* spp. are Gram-negative rods found ubiquitously in the environment, with certain species being reported as having unusual degrading properties. Polyvinyl alcohol (PVA) is used widely in industry but causes serious global environmental pollution. Here, we report the complete genome sequence of a novel bacterium, AC3, that efficiently degrades PVA. As the representative genome of Chryseobacterium nepalense, key genomic characteristics (e.g., mobile genetic elements, horizontal genes, genome-scale metabolic network, secondary metabolite biosynthesis gene clusters, and carbohydrate-active enzymes) were comprehensively investigated to reveal the potential genetic features of this species. Core genome phylogenetic analysis in combination with average nucleotide identity, average amino acid identity, and *in silico* DNA-DNA hybridization values provided an accurate taxonomic position of *C. nepalense* in the genus *Chryseobacterium*. Comparative genomic analysis of AC3 with closely related species suggested evolutionary dynamics characterized by a species-specific genetic repertoire, dramatic rearrangements, and evolutionary constraints driven by selective pressure, which facilitated the speciation and adaptative evolution of *C. nepalense*. Biogeographic characterization indicated that this species is ubiquitously distributed not only in soil habitats but also in a variety of other source niches. Bioinformatic analysis revealed the potential genetic basis of PVA degradation in AC3, which included six putative genes associated with the synthesis of PVA dehydrogenase, cytochrome *c*, oxidized PVA hydrolase, and secondary alcohol dehydrogenase. Our study reports the first complete genome of *C. nepalense* with PVA-degrading properties, providing comprehensive insights into the genomic characteristics of this species and increasing our understanding of the microbial degradation of PVA.

**IMPORTANCE** Although PVA is a biodegradable polymer, the widespread use of PVA in global industrialization has resulted in serious environmental problems. To date, knowledge of effective and applicable PVA-degrading bacteria is limited, and thus, the discovery of novel PVA biodegraders is pertinent. Here, we isolated a novel bacterial strain, AC3, which efficiently degraded PVA. The complete genome of AC3 was sequenced as the first genome sequence of the species *C. nepalense*. Comparative genomic analysis was performed to comprehensively investigate the phylogenetic relationships, genome-scale metabolic network, key genomic characteristics associated with genomic evolution, evolutionary dynamics between AC3 and its close relatives, and biogeographic characterization of *C. nepalense*, particularly regarding the potential genetic basis of PVA degradation. These findings could advance our understanding of the genomic characteristics of *C. nepalense* and PVA bioremediation.

## INTRODUCTION

The genus *Chryseobacterium*, as a member of the family *Flavobacteriaceae* belonging to the phylum *Bacteroidetes*, was first proposed by Vandamme et al. (1) and subsequently emended by Nguyen et al. (2), Montero-Calasanz et al. (3), Hahnke et al. (4), and Nicholson et al. (5). At present, this genus comprises 133 species with validly published names recognized by the LPSN database (accessed on 20 May 2022) (6). The species of the genus *Chryseobacterium* are typically characterized as being rod-shaped, Gram-negative, nonsporulating, nonmotile, and aerobic and as producing nondiffusible flexirubin-type pigments. The members of this genus have been isolated from a wide variety of habitats, including plants, soil, the rhizosphere, compost, sludge, sediments, freshwater, wastewater, clinical specimens, animal hosts, and dairy products (7–9). The species Chryseobacterium nepalense was first described by Chaudhary and Kim (10), and the type strain, C-5-3, was isolated from an oil-contaminated soil sample from Biratnagar, Morang, Nepal, during a study of hydrocarbon-degrading bacteria (8). Analysis based on 16S rRNA sequences and DNA-DNA relatedness revealed the presence of *C. nepalense* (8).

With the increased use of plastic, plastic waste has gradually accumulated in the environment, creating a serious ecological problem. Global plastic production has doubled in the last 25 years, and the total weight of all plastic products on the planet is expected to exceed 33 billion tons in 30 years (11). When it is turned into plastic waste, these characteristics pose a major challenge to the degradation of plastic. Recycled plastics are more economically expensive than virgin plastic products, and the quality and performance of recycled plastic products are far inferior to those of new products, a phenomenon known as “down-cycling” (12). The degradation of these stubborn and recalcitrant polymers by microorganisms to achieve harmless or minimally harmful products has been the focus of recent scientific research (13). Polyvinyl alcohol (PVA) is a water-soluble plastic that is marketed as a new plastic alternative that is “biodegradable.” PVA is widely used in the fields of catering, textiles, paper, printing, and packaging (14, 15). Due to the high volume of production and utilization, significant accumulation of PVA in the environment has been detected worldwide (16). Although biodegradable, the degradation of PVA in the natural environment is a slow process (17). Compared to other hydrophobic plastics, PVA poses a more serious hazard to water sources. Once it is dissolved in water, it is difficult to remove PVA from contaminated water unless it undergoes natural degradation over a long period of time (18). Due to its hydrophilicity, PVA that accumulates in the environment can more easily adsorb antibiotics and heavy metals than other traditional plastics (19). Microbial degradation is considered a promising alternative for effective PVA removal. Most studies on PVA-degrading bacteria have mainly focused on some well-studied microorganisms, including Pseudomonas, *Sphingomonas*, and *Stenotrophomonas* (20–22), of which only *Sphingopyxis* sp. strain 113P3 and Stenotrophomonas rhizophila QL-P4 have been sequenced. Thus, acquiring further genome data for a novel PVA-degrading bacterium could expand our understanding of PVA bioremediation.

In this study, we report a novel strain, AC3, which was isolated from plastic-attached soil samples and identified as the species *C. nepalense*. Whole-genome sequencing (WGS) offers a tremendous advantage in determining evolutionary relationships, genomic characteristics, and biotechnological properties (23–25). Hence, we present the complete genome sequence of AC3. Comparative genomic analysis was performed to explore the phylogenetic relationships, genome-scale metabolic networks, key genomic characteristics associated with genomic evolution, evolutionary dynamics between AC3 and closely related species, and biogeographic characterization of *C. nepalense*. These findings enhance our understanding of the species *C. nepalense* at the whole-genome level. Furthermore, we reported that strain AC3 has the ability to degrade PVA, and we subsequently explored the putative genotypic profile for PVA degradation in the AC3 genome. This is the first report of a *Chryseobacterium* strain with PVA-degrading properties, thereby increasing our understanding of its potential for the bioremediation and microbial degradation of PVA.

## RESULTS AND DISCUSSION

### Isolation, identification, and characterization of AC3.

**FIG 1:**
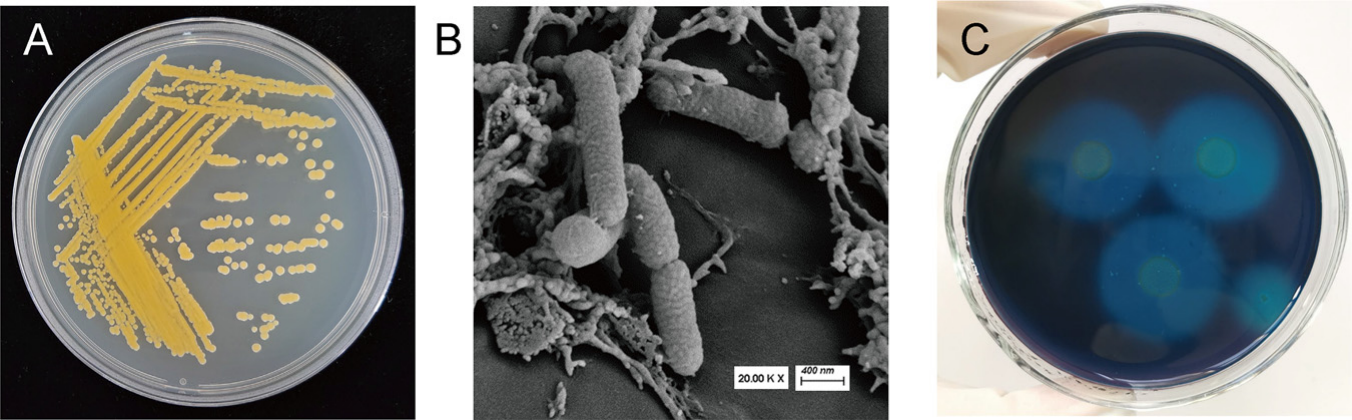
Phenotypic characteristics of strain AC3. (A) Morphological characteristics of the colony. Strain AC3 is orange, and the colonies are moist, rounded, nonsporulating, nonmotile, and aerobic. (B) Scanning electron micrograph showing the morphology of strain AC3. The bacterium is rod shaped and has a pitted surface. (C) PVA screening medium with the zone of clearance around the colonies of AC3, after pouring with an iodine-boric acid solution.

Strain AC3 was isolated from the plastic-attached soil on the south side of Mount Tai in China (geographic coordinates 36°20 N, 117°13 E). Strain AC3 on Luria-Bertani (LB) medium is orange and produces an orange pigment; the colonies are moist, rounded, nonsporulating, nonmotile, and aerobic (Fig. 1A). Strain AC3 was observed under a scanning electron microscope, and the bacterium is rod shaped and has a pitted surface (Fig. 1B). The growth curve of strain AC3 on LB liquid culture medium consists of a short lag phase followed by logarithmic growth, and then a stationary phase as the bacteria form a confluent monolayer (see Fig. S1 in the supplemental material). Strain AC3 demonstrated PVA-degrading abilities on the screening agar medium using PVA as the sole carbon source when clear zones developed around the colony after being flooded with iodine-boric acid solution (Fig. 1C and Fig. S2A). This strain could also degrade PVA in the liquid medium with PVA as the sole carbon source. In this case, cell growth was observed (Fig. S2B). Meanwhile, it did not show any growth in the absence of PVA in the liquid medium (Fig. S2C). Considering these characteristics, strain AC3 is expected to degrade PVA.

### Genomic characteristics of AC3.

**FIG 2:**
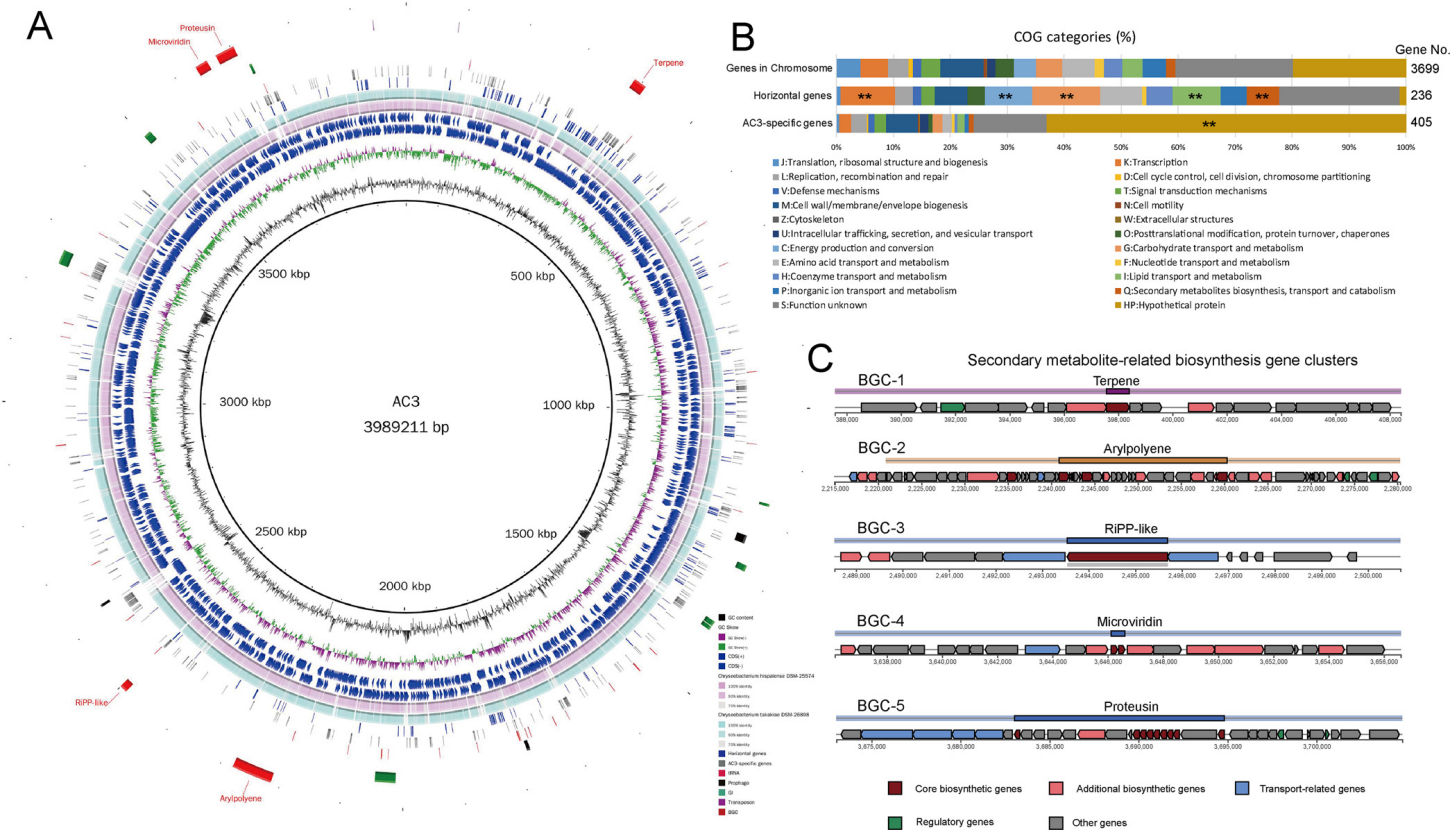
Genome features of the AC3 genome. (A) Circular representation of the AC3 genome. Rings represent the following features, from the inside to the outside: ring 1, GC content; ring 2, GC skew (green and purple correspond to above- and below-average GC skew, respectively); rings 3 and 4, blue arrows correspond to plus-strand CDS and minus-stand CDS, respectively; rings 5 and 6, circular comparison of Chryseobacterium hispalense DSM 25574 and Chryseobacterium takakiae DSM 26898, respectively; rings 7 to 13, blocks correspond to potential horizontal genes, AC3-specific genes, tRNA, prophage, GIs, transposon, and secondary metabolite-related biosynthesis gene clusters, respectively. (B) Distribution of COG categories for chromosome genes, AC3-specific genes, and potential horizontal genes. **, *P* < 0.01 (Fisher’s exact test). (C) Genetic organization of the gene clusters for secondary metabolism in the AC3 genome.

To explore the genomic evolution and underlying biotechnological properties of AC3, the complete genome of AC3 was sequenced. The AC3 genome is composed of one circular chromosome. No plasmid was detected. The genome was estimated to be 100% complete with less than 0.5% contamination. The genomic size was 3,989,211 bp, with an average GC content of 36.9% and 3,699 RAST-predicted coding sequences (CDSs) (Table S1). A total of 80 RNA sequences were identified in the AC3 genome, including 15 rRNA genes (5S, 5 genes; 16S, 5 genes; 23S, 5 genes) and 65 tRNA genes. The general genomic properties of AC3 are shown in Fig. 2A.

Based on the Cluster of Orthologous Groups (COG) functional annotation, a total of 2,921 (79.0%) CDSs were identified and classified into 20 COG functional categories (Fig. 2B), mainly including “M: Cell wall/membrane/envelope biogenesis” (299 CDSs), “E: Amino acid transport and metabolism” (225 CDSs), “K: Transcription” (188 CDSs), “G: Carbohydrate transport and metabolism” (177 CDSs), and “J: Translation, ribosomal structure and biogenesis” (167 CDSs). Furthermore, there are 778 CDSs with no homologs in the COG database. In this study, these CDSs were designated as functional category “HP: Hypothetical protein.” A total of 1,587 CDSs were poorly characterized (“S: Function unknown,” 809 CDSs; “HP: Hypothetical protein,” 778 CDSs), making up 42.9% of the gene repertoires. Therefore, the high proportion of poorly functional CDSs distributed in the AC3 genome requires further research.

Secondary metabolites are versatile compounds that bacteria use to fight other microbes (26). We also identified five putative secondary metabolite biosynthesis gene clusters (BGCs) involved in the synthesis of terpene (BGC-1), aryl polyene (BGC-2), RiPP-like peptide (BGC-3), microviridin (BGC-4), and proteusin (BGC-5) (Fig. 2C and Table S2). All BGCs in AC3 showed high homology to gene clusters from other *Chryseobacterium* strains (Fig. S3). By using a MIBiG (minimum information about a biosynthetic gene cluster) comparison, 77% of the genes of BGC-2 were found to be homologous to those of the known BGC of flexirubin. BGC-1 contained a fairly low proportion of genes homologous to the BGC of carotenoids. BGC-3, -4, and -5 did not show similarities to any known BGCs present in the antiSMASH database.

### Phylogenetic and genomic analysis resolves the evolutionary position of AC3.

**FIG 3:**
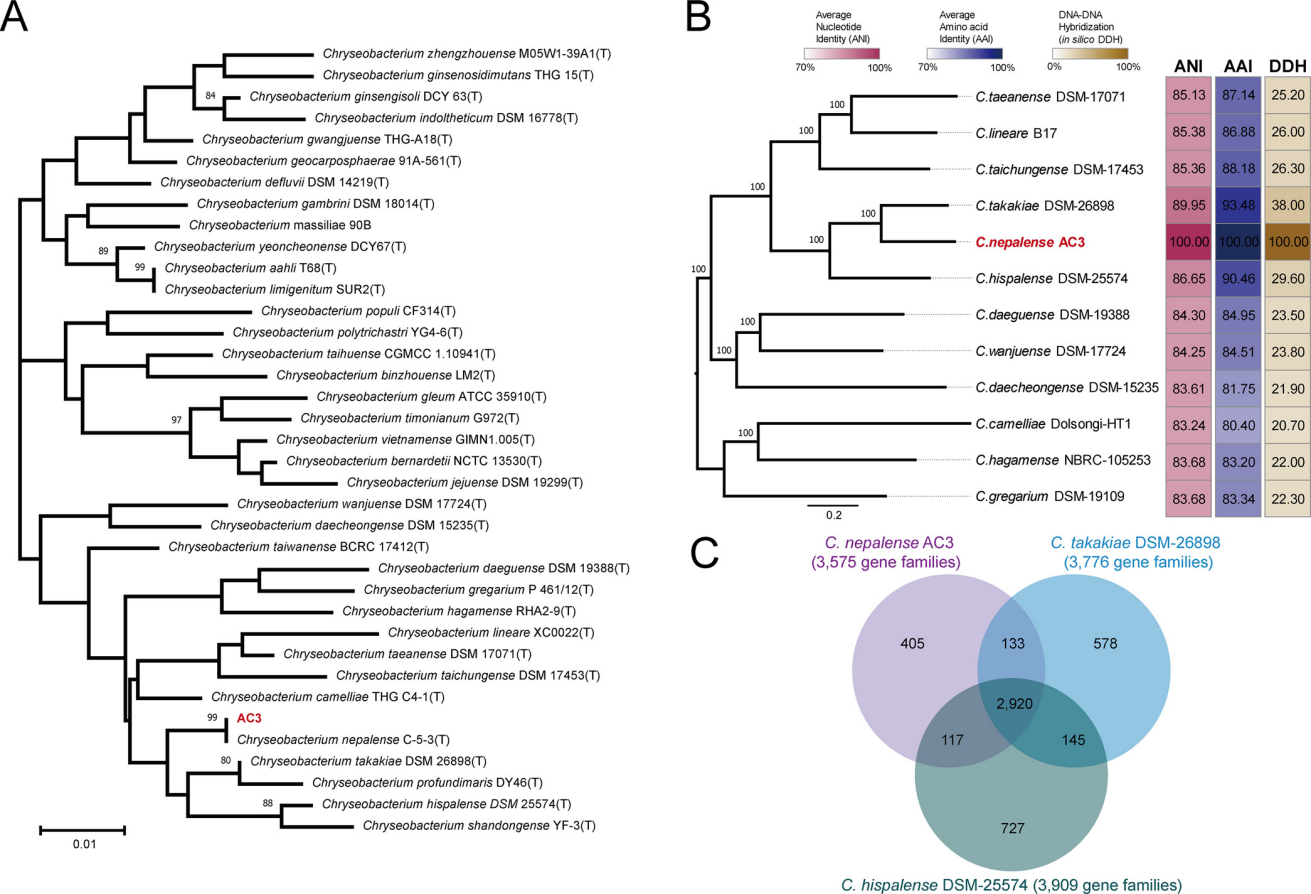
Phylogenetic analysis and genomic characteristics of AC3 belonging to the genus *Chryseobacterium*. (A) Phylogenetic tree based on 16S rRNA sequences obtained by the ML method. The node values of the tree are bootstrap values (1,000 replicates). Numbers at nodes indicate the levels of bootstrap support (>70%). (B) Phylogenetic tree based on SNPs across 1,833 single-copy core gene families shared by the AC3 genome and the other 11 *Chryseobacterium* genomes constructed by the ML method with 100 replicates. The values next to the tree indicate ANI, AAI, and *in silico* DDH values. The color blocks present ANI (navy), AAI (maroon), and *in silico* DDH (brown). (C) Venn diagram showing overlaps and differences in orthologous gene families in AC3 and closely related strains (*C. hispalense* DSM 25574 and *C. takakiae* DSM 26898).

To determine the evolutionary position of AC3, we performed a phylogenetic and genomic analysis based on whole-genome data. First, the 16S rRNA sequence (1,523 bp with 100.0% completeness) of AC3 was subjected to similarity-based searches against the taxonomically united 16S rRNA database in EzBioCloud (27) to identify potential closely related species. Then, the 16S rRNA sequences of closely related species were collected from the EzBioCloud database (Table S2) and used to construct a phylogenetic tree using the maximum-likelihood (ML) method. As shown in Fig. 3A, strain AC3 together with Chryseobacterium nepalense C-5-3^T^ clustered in a separate clade within the genus *Chryseobacterium.* Meanwhile, the 16S rRNA sequence of AC3 showed 100.0% similarity with the corresponding genes from *C. nepalense* C-5-3^T^ (Table S2). Thus, our results indicate that AC3 may be a member of the species *C. nepalense*.

To further evaluate the evolutionary position of AC3 at the whole-genome level, 11 reference genomes of the closest related *Chryseobacterium* species on the 16S tree (Fig. 3A and Table S3) were collected in combination with the AC3 genome for subsequent analyses. An ML tree was constructed based on the single-nucleotide variants for the 1,833 single-copy core gene families shared by all 12 genomes. The average nucleotide identity (ANI), average amino acid identity (AAI), and *in silico* DNA-DNA hybridization (DDH) values were also calculated to measure the genetic relatedness between AC3 and other members of *Chryseobacterium*. In the core genome tree, AC3 together with Chryseobacterium takakiae DSM 26898 and Chryseobacterium hispalense DSM 25574 formed a monophyletic clade (Fig. 3B), indicating a close evolutionary relationship. The ANI and AAI values determined from comparisons between AC3 and other members of *Chryseobacterium* were 83.2% (Chryseobacterium camelliae Dolsongi-HT1) to 90.0% (*C. takakiae* DSM 26898) and 80.4% (*C. camelliae* Dolsongi-HT1) to 93.5% (*C. takakiae* DSM 26898) (Fig. 3B), respectively, which were lower than the 95% threshold value for species delimitation (28). The *in silico* DDH value shared by AC3 and other genomes was 20.7% (*C. camelliae* Dolsongi-HT1) to 37.3% (*C. takakiae* DSM 26898) (Fig. 3B), which was also much lower than the 70% threshold value for species delimitation (29). On these bases, strain AC3 can be recognized as a novel strain of the species *C. nepalense*.

### Genome-scale metabolic network construction for AC3 provides insights into the metabolic capacity of *C. nepalense*.

**FIG 4:**
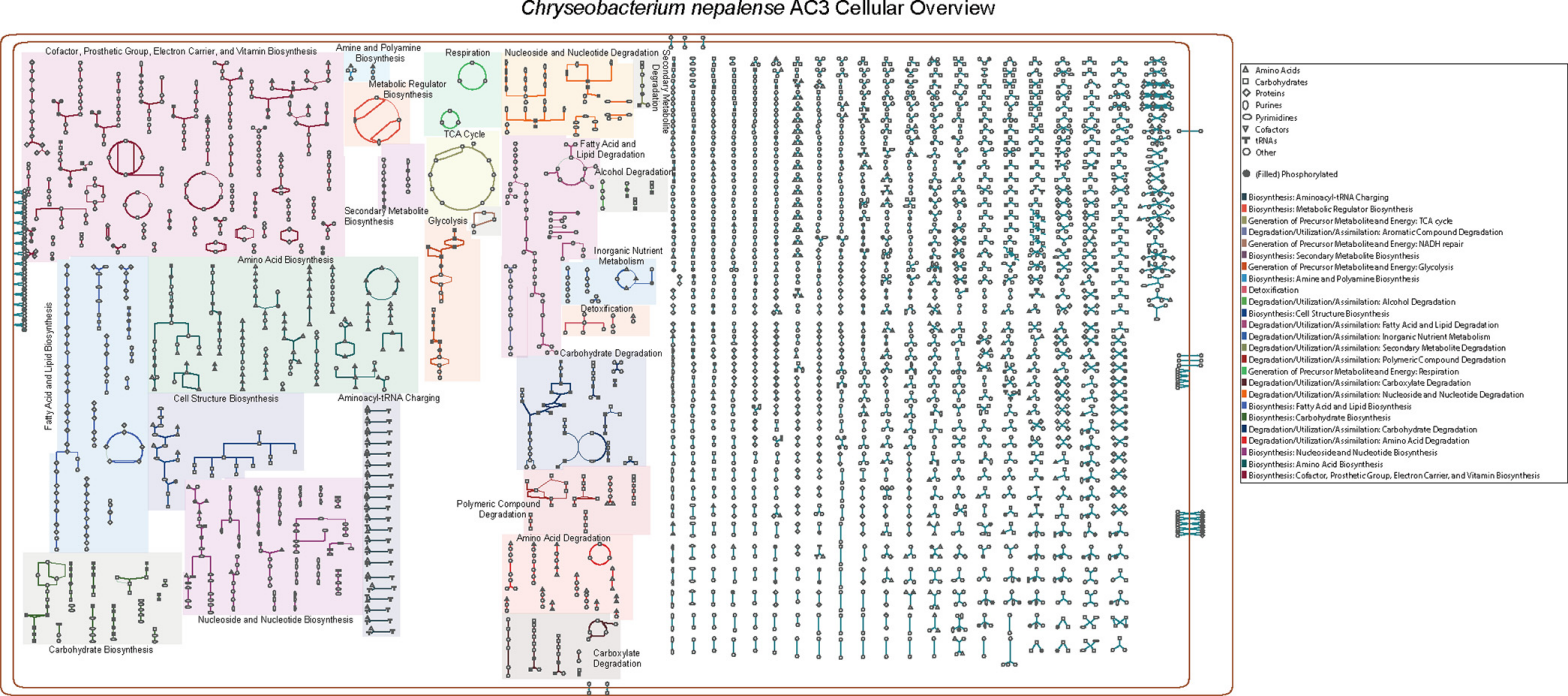
Complete components of the genome-scale metabolic network for AC3. Symbols represent metabolites, and connecting lines represent enzymes catalyzing the corresponding reactions. Brown lines represent the two cell membranes.

As the first complete genome for the species *C. nepalense*, the construction of the metabolic network based on the AC3 genome could provide insights into the metabolic capacity of *C. nepalense*. The automatically generated pathway/genome database (PGDB) for the AC3 functional genome-scale metabolic network is composed of 829 enzymes, 13 transporters, and 1,332 metabolites organized into 210 pathways with a total of 1,634 reactions, including 10 transport reactions and 1,624 enzymatic reactions. Figure 4 shows an overview of the genome-scale metabolic network, in which nodes represent metabolites and links represent the reactions. AC3 has a central metabolic repertoire with capabilities of glycolysis, pyruvate decarboxylation to acetyl coenzyme A (acetyl-CoA), and tricarboxylic acid (TCA) cycle. As an aerobic bacterium, four pathways for aerobic respiration and no anaerobic respiration pathway were identified in the AC3 genome, namely, NADH-to-cytochrome *bd* oxidase electron transfer I (PWY0-1334), NADH-to-cytochrome *bd* oxidase electron transfer II (PWY0-1568), pyruvate-to-cytochrome *bd* oxidase electron transfer (PWY-7545), and succinate-to-cytochrome *bd* oxidase electron transfer (PWY0-1353). These respiratory chains formed by dehydrogenases and cytochrome *bd* oxidase (encoded by CDS3655 and CDS3656) transferred electrons from NADH, pyruvate, and succinate to oxygen to generate a proton-motive force across the cytoplasmic membrane. Under conditions of aerobic growth, pyruvate and succinate were largely derived from glycolysis and the TCA cycle, respectively. However, during glucose-limited aerobic growth, AC3 could direct electron flux through both NADH dehydrogenase I (NDH-I) and NDH-II (30). The set of anabolic pathways was involved in the biosynthesis of amino acids, fatty acids, phospholipids, nucleotides, vitamins, cofactors, putrescine, spermidine, and secondary metabolites. Additionally, 21 aminoacyl-tRNA charging metabolic clusters were identified.

Abundant reactions and metabolites belonged to degradation-related pathways for nucleoside, nucleotide, fatty acid, lipid, carbohydrate, alcohol, amino acid, carboxylate, and polymeric compounds, as well as for secondary metabolites, indicating the metabolic potential of AC3 biodegradation (Fig. 4). The AC3 genome also harbored several detoxification-related pathways, including arginine-dependent acid resistance (PWY0-1299), arsenate detoxification II (PWY-4621), and reactive oxygen species degradation (DETOX1-PWY-1). These characteristics could protect the host microorganism against the harmful effects of toxic conditions and contribute to the stress resistance and niche adaptation of AC3. Additionally, a significant number of reactions or metabolites in parts of the overall metabolic network are still not associated with any metabolic pathway category (Fig. 4). The potential biological function of these limited known metabolic reactions and metabolites should be an important focus in further functional studies to enable us to fully exploit the metabolic capacity of *C. nepalense*.

### Genomic evolution of AC3 exhibited by MGEs and potential horizontal genes.

Mobile genetic elements (MGEs) can mediate the transmission of genetic material and facilitate bacterial genomic evolution (31, 32). Several types of MGEs were identified in the AC3 genome, including prophages, genomic islands (GIs), and potential transposons (Fig. 2A and Table S4). Three incomplete prophages were identified, covering 24.9 kb of the genomic region. These prophage regions are similar to PHAGE_Acidia_virus (NC_029316), PHAGE_Caulob_CcrPW (NC_048046), and PHAGE_Bacill_G (NC_023719). The AC3 genome harbored eight GIs that occupied a 107.4-kb region of the genome. Two transposons (CDS79 and CDS3643) were detected in AC3 using ISfinder software (33). CDS79 and CDS3643 were most homologous to ISCysp14 and IS609, with coverages of 76.2% and 73.0%, identities of 30.5% and 26.5%, and E values of 1.64e−11 and 9.68e−16, respectively. Thus, these two transposons may be novel ISs.

Horizontal gene transfer (HGT) mediated by MGEs is the major driver of bacterial genomic evolution and promotes the acquisition of new physiological functions, which is crucial for niche adaptation (34, 35). A total of 236 potential horizontal genes were identified (Table S5). Based on the COG annotation, these horizontal genes were significantly involved in “K: Transcription” and in several metabolism-related categories, including “C: Energy production and conversion,” “G: Carbohydrate transport and metabolism,” “I: Lipid transport and metabolism,” and “Q: Secondary metabolites biosynthesis, transport and catabolism” (Fisher’s exact test *P* value < 0.05) (Fig. 2B). It can be inferred that the acquisition of the novel metabolism-related genetic properties driven by HGT promoted the adaptation of AC3 to diverse soil environments. In addition, several genetic elements have been proposed as HGT barriers to control bacterial genomic stability (36, 37), including restriction-modification (RM) systems, toxin/antitoxin (TA) systems, and clustered regularly interspaced palindromic repeats (CRISPR). However, no such barrier to HGT was detected in the AC3 genome. The absence of HGT barriers combined with the presence of MGEs and horizontally transferred genes indicated the genomic plasticity of AC3.

### Comparative genomic analysis of AC3 with closely related species revealed its evolutionary dynamics.

The exploration of the evolutionary dynamics of the AC3 genome could provide insights into the speciation of *C. nepalense*. Hence, we conducted a comparative genomic analysis using AC3 for *C. nepalense*, DSM 26898^T^ for *C. takakiae*, and DSM 25574^T^ for *C. hispalense*. We found 2,920 gene families shared among all three genomes. A total of 405 gene families were identified as AC3-specific gene repertoire, which were present in the AC3 genome and absent in the *C. takakiae* DSM 26898 and *C. hispalense* DSM 25574 genomes (Fig. 3C). Additional features in some AC3-specific genes, such as adjacent tRNAs and transposons, as well as remnants of GIs and prophages, indicated the occurrence of HGT in the AC3 genome (Fig. 2A). Therefore, HGT mediated by MGEs, as important evolutionary forces, contributed to the genomic evolution and speciation of *C. nepalense*. These AC3-specific gene families were mainly composed of “HP: Hypothetical protein” (Fisher’s exact test *P* value < 0.01)-encoding genes (Fig. 2B). Meanwhile, they were also involved in three MetaCyc metabolic pathways, namely, d-gluconate degradation (GLUCONSUPER-PWY), acyl carrier protein metabolism (PWY-6012), and chitin degradation III (PWY-7822) (Fig. S4). These findings indicated the differentiation in metabolic abilities and niche adaptation between AC3 and closely related species.

**FIG 5:**
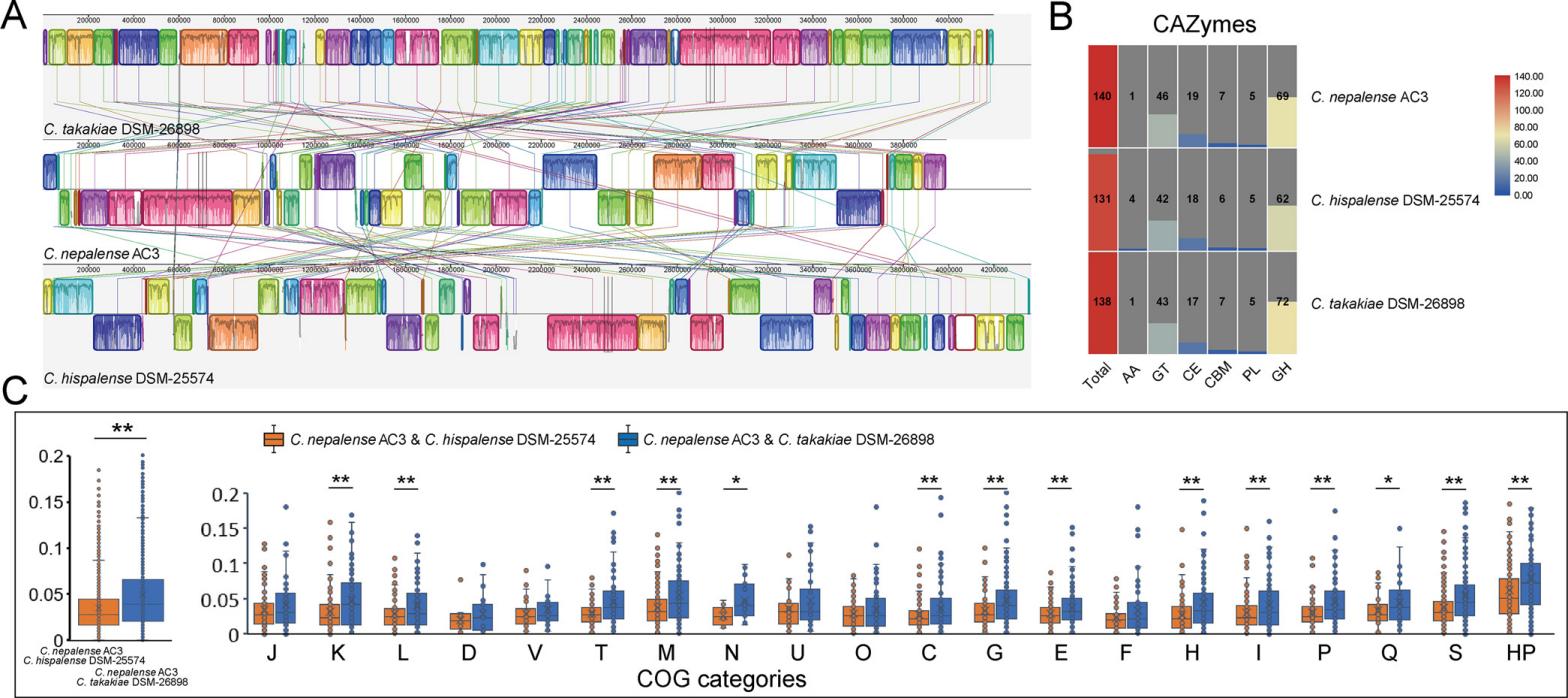
Comparative genomic analysis of AC3 and closely related strains. (A) Genome alignment of AC3, *C. hispalense* DSM 25574, and *C. takakiae* DSM 26898. Synteny blocks are shown as identically colored regions and are linked across the sequences. (B) Distribution of CAZymes in AC3 and closely related strains. (C) Comparisons of the synonymous (*Ka*)/nonsynonymous (*Ks*) substitution rates of the orthologous mutational gene pairs between AC3 and *C. hispalense* DSM 25574 and between AC3 and *C. takakiae* DSM 26898. *, *P* < 0.05; **, *P* < 0.01 (*t* test).

We performed synteny analysis to provide insights into the genomic dynamics. The synteny comparison of the genomes exhibited a high level of synteny with rearrangements, inversions, and deletions (Fig. 5A). A total of 57 synteny blocks was identified, spanning 3,760,456 bp (94.3%), 3,840,885 bp (91.5%), and 3,761,965 bp (86.2%) in AC3, *C. takakiae* DSM 26898, and *C. hispalense* DSM 25574, respectively. These synteny blocks were considerably fragmented, and most of these blocks were not long. Carbohydrate-active enzymes (CAZymes) are the most important enzymes for complex carbohydrate metabolism. The AC3 genome harbored 140 CAZyme-encoding genes, distributed in 61 CAZyme families (Fig. 5B). Glycoside hydrolases (GHs; *n *= 69; 49.3%) and glycosyltransferases (GTs; *n *= 46; 32.9%) families showed high proportions among CAZymes. The other CAZymes were carbohydrate esterases (CEs; *n* = 19), carbohydrate binding molecules (CBMs; *n *= 7), polysaccharide lyases (PLs; *n *= 5), and auxiliary activity (AA; *n *= 1). The comparison of the CAZyme repertoire of AC3 with closely related species indicated that AC3 harbored seven strain-specific CAZyme-encoding genes for four GTs and three GHs, namely, CDS341 (GH3), CDS345 (GH3), CDS1063 (GH130), CDS1064 (GT4), CDS1902 (GT2), CDS1904 (GT2), and CDS3157 (GT2). These specific CAZyme-encoding genes might contribute to the metabolic adaptation of AC3 to diverse environments, particularly in municipal-waste-contaminated soil.

### Selective pressure analysis revealed the dynamics of natural selection occurring between AC3 and closely related species.

The evolutionary dynamics could be further exhibited by the level of genetic divergence and signatures of natural selection between AC3 and closely related species. We calculated average nonsynonymous (*Ka*) and synonymous (*Ks*) substitution rates (*Ka*/*Ks*) for strain pairs using 2,339 reciprocal best hits (RBH) gene pairs for AC3 and *C. takakiae* DSM 26898 and 1,972 RBH gene pairs for AC3 and *C. hispalense* DSM 25574. Overall, the *Ka*/*Ks* rates of all gene pairs were less than 1, exhibiting a predominant action of purifying selection. Interestingly, although AC3 exhibited a closer relationship to *C. takakiae* DSM 26898 than *C. hispalense* DSM 25574 in the core genome tree (Fig. 3B), AC3 and *C. takakiae* DSM 26898 seemed to undergo more highly divergent evolution affected by natural selection. As shown in Fig. 5C, the gene pairs between AC3 and *C. hispalense* DSM 25574 (average *Ka*/*Ks* rates = 0.0351 ± 0.0310) were under significantly stronger purifying selection than the gene pairs between AC3 and *C. takakiae* DSM 26898 (average *Ka*/*Ks* rates = 0.0495 ± 0.0429; *t* test, *P < *0.01) (Fig. 5C). It can be assumed that the significant genetic divergence between strain pairs revealed the adaptive evolution driven by the difference in the habitats of these strains. Indeed, strain AC3 was isolated from municipal-waste-contaminated soil, whereas *C. hispalense* DSM 25574 was isolated from a rainwater pond in an olive plant nursery (38). However, *C. takakiae* DSM 26898, as an endophytic bacterium, was isolated from the host plant (a type of bryophyte Takakia lepidozioides) (39). Due to the different niches between AC3 and *C. takakiae* DSM 26898, the weaker purifying selection could promote adaptation during habitat conversion. In contrast, AC3 and *C. hispalense* DSM 25574 were isolated from similar niches, resulting in a stronger tendency to keep conserving basic functions.

We further investigated the evolutionary dynamics in each functional category. The significant divergences of purifying selection between strain pairs were observed in most functional categories (*n *= 14) (Fig. 5C). For instance, the evolutionary constraints of the gene pairs between AC3 and *C. takakiae* DSM 26898 associated with “Information Storage and Processing” (K: Transcription; L: Replication, recombination and repair) and “Cellular Processes and Signaling” (T: Signal transduction mechanisms; M: Cell wall/membrane/envelope biogenesis; N: Cell motility) were significantly weaker than those of the gene pairs between AC3 and *C. hispalense* DSM 25574 (*t* test, *P < *0.05). Notably, the gene pairs between AC3 and *C. takakiae* DSM 26898 underwent significantly weaker evolutionary constraints in most functional categories of “Metabolism” than the gene pairs between AC3 and *C. hispalense* DSM 25574 (*t* test, *P < *0.05), except for “F: Nucleotide transport and metabolism” (Fig. 5C).

### Global distribution and abundance of *C. nepalense*.

**FIG 6:**
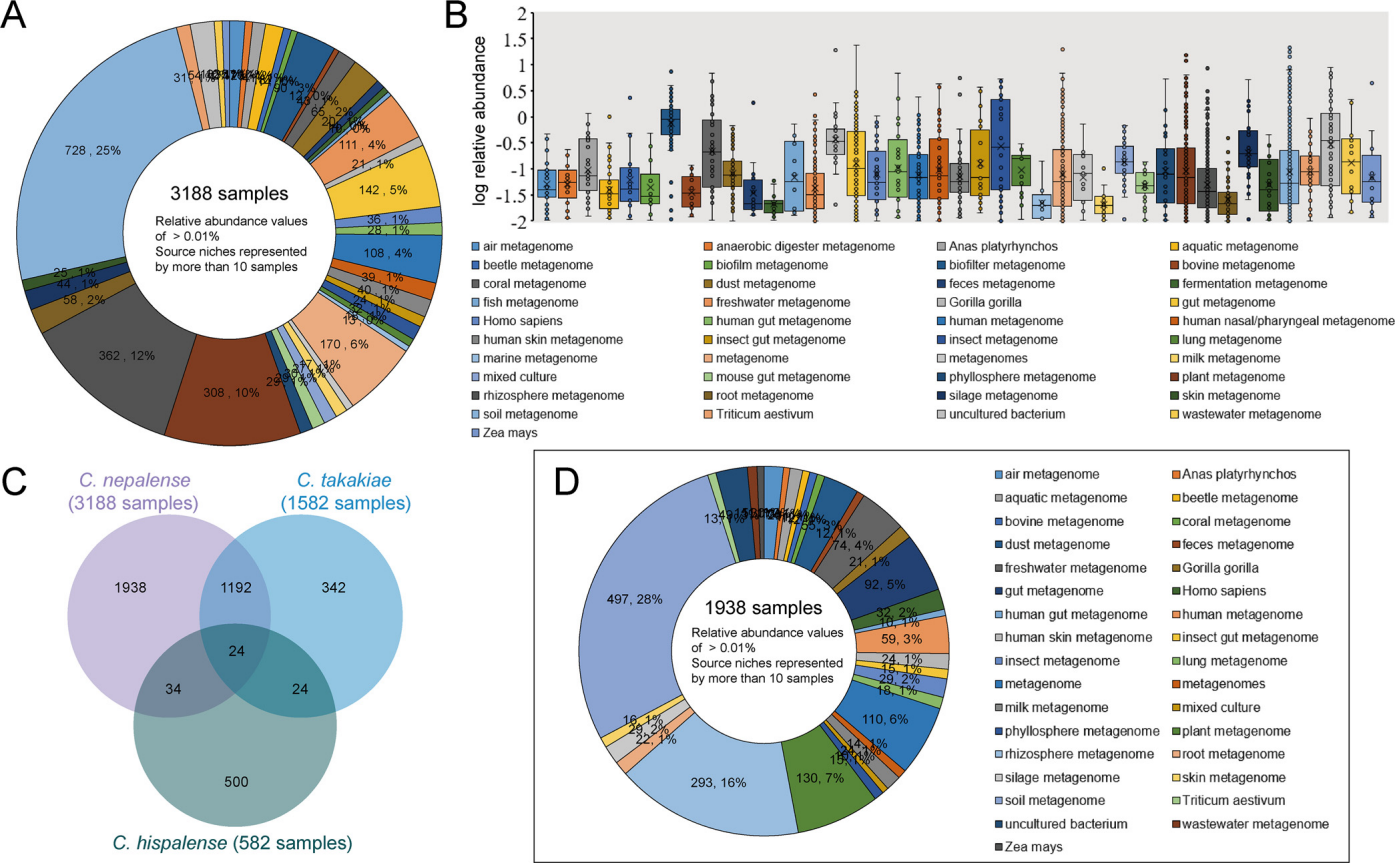
Biogeographic distribution and relative abundance profiles of *C. nepalense*. (A) Distribution of environmental sources that contain operational taxonomic units affiliated with *C. nepalense*. For better readability, source niches represented by fewer than 10 samples screened by the IMNGS platform and relative abundance values of ≤0.01% are not plotted. Colors correspond to source niches. (B) Relative abundances of the 16S rRNA sequence of *C. nepalense* in different source niches. (C) Venn diagram displaying overlaps and differences in environmental samples in *C. nepalense* and closely related species. (D) Distribution of environmental sources contain only operational taxonomic units affiliated with *C. nepalense* and not with *C. takakiae* and *C. hispalense*. Colors correspond to source niches.

*Chryseobacterium* sp. is ubiquitously distributed in the environment. Various strains have been isolated from soil, plants, waste, sewage, sludge, lactic acid beverages, oil-contaminated soil, and clinical samples (2, 40). To investigate the potential biogeographical distribution of *C. nepalense*, we queried the representative 16S rRNA sequence (GenBank accession no. KX129820) of *C. nepalense* against all publicly available (*n *= 500,048; accessed 10 April 2022) SRA data sets using the Integrated Microbial Next Generation Sequencing (IMNGS) platform. The target 16S rRNA sequence of *C. nepalense* was detected in 5,559 samples collected from 187 categories of source niches (Table S6), indicating ubiquitous members of *C. nepalense* that have been isolated and identified from almost all screened environments (e.g., soil, rhizosphere, plant, freshwater, dust, aquatic, air, insects, and animal guts). Among these, 3,188 samples exhibited >0.01% relative abundances, which were collected from 124 categories of source niches (Fig. 6A), mainly including “soil metagenome” (*n *= 728; average relative abundance = 0.659% ± 0.0526%), “rhizosphere metagenome” (*n *= 362; average relative abundance = 0.161% ± 0.00298%), and “plant metagenome” (*n *= 308; average relative abundance = 0.606% ± 0.0317%) (Fig. 6B). The four samples where the *C. nepalense* 16S rRNA sequence had substantial abundance accounted for more than 20% of the total community and consisted of SRR2848934 (24.632%; soil metagenome associated with weed seed), SRR3493009 (23.788%; gut metagenome of Salamandra salamandra), SRR8655949 (21.939%; soil metagenome from methane and methanethiol enriched culture), and SRR8663214 (21.482%; metagenome from a diesel-degrading soil bacterial consortium growing with different substrates) (Table S6). These findings indicated that the *C. nepalense* strains might be unequally distributed in diverse source niches.

Additionally, the representative 16S rRNA sequences of *C. takakiae* and *C. hispalense* were present only in 2,676 and 1,290 samples collected from 137 and 110 categories of source niches, of which 1,528 and 582 samples exhibited >0.01% relative abundances, respectively. Most of the samples (*n* = 1,938; relative abundances > 0.01%) harboring the *C. nepalense* members had no sequences related to the *C. takakiae* or *C. hispalense* strains (Fig. 6C). As shown in Fig. 6C, *C. nepalense* was almost unique to soil (*n *= 497)-, rhizosphere (*n *= 293)-, and plant (*n *= 130)-related samples. Our results indicated that *C. nepalense* seemed to have a broader and more distinct environmental distribution than other closely related species.

### Potential genetic basis of PVA degradation in the AC3 genome.

**FIG 7:**
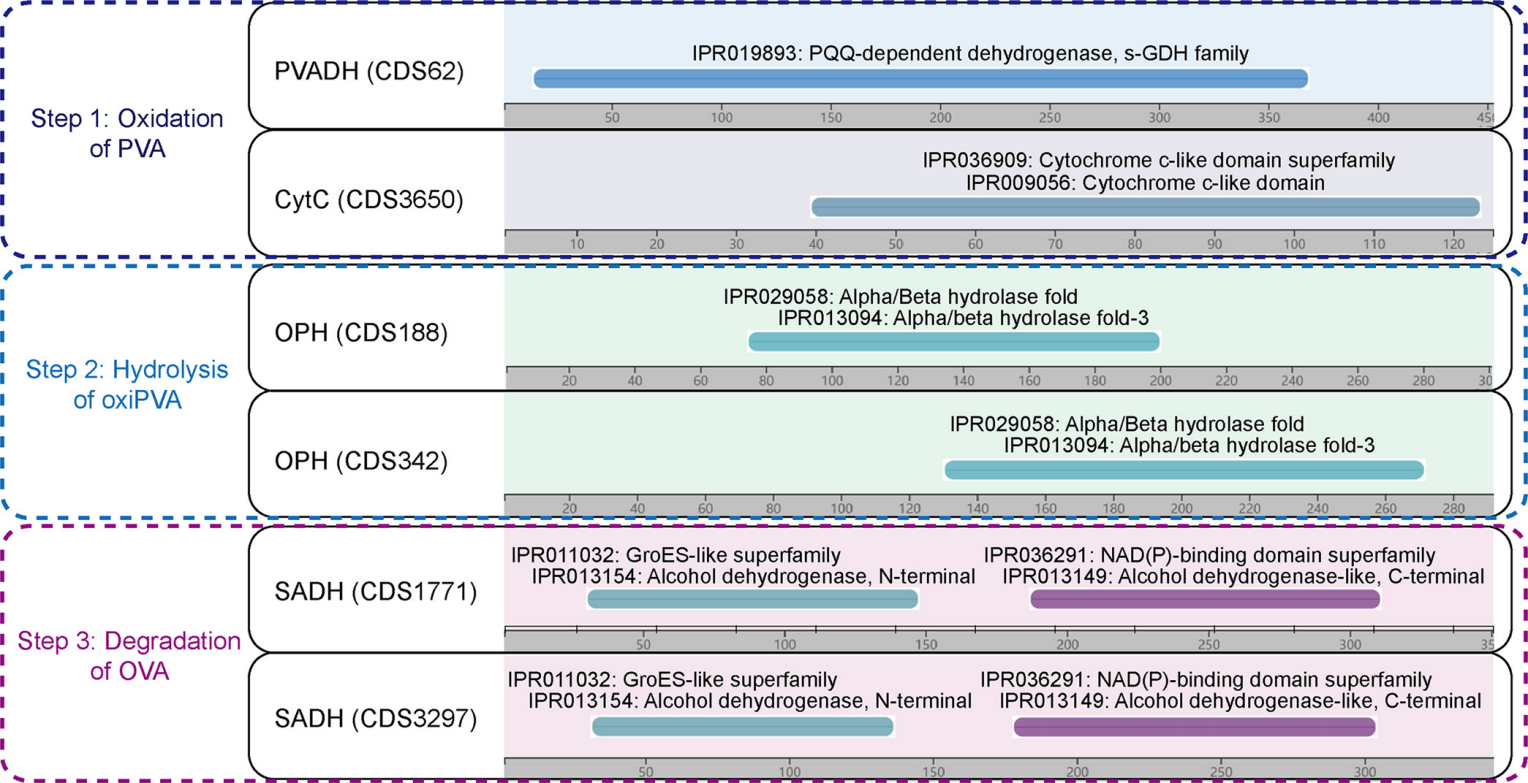
Six genes in the AC3 genome predicted to participate in PVA degradation. Identification of putative PVA-degrading enzymes was performed using domain comparison. Domains were searched against the InterPro database and are shown as different-colored bars.

The PVA chemical structure mainly comprises repeated 1,3-diol units that can be degraded to a vinyl alcohol oligomer (OVA) by microorganisms in a two-step reaction (oxidation and hydrolysis). The oxidation may occur via a secondary alcohol oxidase (SAO) using oxygen as an electron acceptor; it also may occur via a periplasmic PVA dehydrogenase (PVADH) whose degradation pathway includes the action of pyrroloquinoline quinone (PQQ) as well as soluble cytochrome *c* (CytC) and oxygen as terminal electron acceptors (16, 41). Subsequently, oxidized PVA (oxiPVA) can be hydrolyzed by oxidized PVA hydrolase (OPH) into OVA, which can then be oxidized by secondary alcohol dehydrogenase (SADH) (20, 42). In this study, the integration of functional annotation, BLASTp search, and corresponding domains reportedly involved in PVA degradation (22) allowed us to identify six putative PVA-degrading genes in the AC3 genome encoding PQQ-dependent dehydrogenase (PVADH; CDS62), CytC (CDS3650), OPH (CDS188 and CDS342), and SADH (CDS1771 and CDS3297) (Fig. 7).

For the oxidation of PVA, the predicted PVADH encoding gene, CDS62, contained a PQQ-dependent dehydrogenase, the s-GDH family (IPR019893) domain. Meanwhile, CDS3650 harboring a cytochrome *c*-like domain (IPR009056) may encode cytochrome *c* as an electron acceptor for PVADH in the oxidation of PVA degradation. In addition, we did not identify any putative SAO genes encoding secondary alcohol oxidases in the AC3 genomes, indicating that the intracellular degradation of PVA with oxidation by PAVDH seems to be a dominant pathway in AC3. For the hydrolysis of oxiPVA, two putative hydrolases encoded by CDS188 and CDS342 shared 38.9% and 28.1% sequence identity, respectively, with the OPH in *S. rhizophila* QL-P4 (22). Both of them contained an alpha/beta hydrolase fold (IPR029058) domain. For the degradation of OVA, two predicted SADH-encoding genes, CDS1771 and CDS3297, shared the same domains as the SADH gene in *S. rhizophila* QL-P4 (22), which included a GroES-like superfamily (IPR011032) domain and a NAD(P)-binding domain superfamily (IPR036291) domain. Overall, the existence of these predicted genes carried by the AC3 genome provides a potential genetic basis for PVA degradation, while the detailed elucidation of how they function requires further transcription and mutation studies in future work.

### Conclusions.

Our study describes the complete genome sequence of a novel aerobic bacterium, AC3, isolated from a plastic-attached soil sample utilizing PVA as the sole source of carbon and energy. This is the first report of a *Chryseobacterium* strain with PVA-degrading properties and the first genome sequence of the species *C. nepalense*. Systematic analysis of the AC3 genome represented key genomic characteristics, including the existence of MGEs, horizontal genes, secondary metabolite biosynthesis, and CAZymes, which revealed the potential genetic features of this species. The phylogenetic analysis based on the core genome in combination with the ANI, AAI, and *in silico* DDH values provides an accurate indication of the taxonomic position as well as the genome characteristics of *C. nepalense* in the genus *Chryseobacterium*. We also constructed a genome-scale metabolic network of AC3 containing degradation-related pathways that can be used to simulate the metabolic capability of *C. nepalense*. Comparative genomic analysis of AC3 with closely related species showed high levels of evolutionary plasticity derived by HGT, with the existence of a species-specific gene repertoire and dramatic rearrangements, which facilitated the speciation and evolution of *C. nepalense*. Different degrees of purifying selection were observed to occur between AC3 and closely related species, which might be the result of niche conversion. Biogeographic characterization indicated that members of this species are found globally in a variety of source niches, such as soil, rhizosphere, plant, freshwater, dust, aquatic, air, and insects, as well as animal guts. Interestingly, *C. nepalense* seems to have a broader and more distinct environmental distribution than the closely related species *C. takakiae* and *C. hispalense*, indicating diverse environmental adaptation. Moreover, we discovered six putative PVA-degrading genes in the AC3 genome encoding PQQ-dependent dehydrogenase (PVADH: CDS62), CytC (CDS3650), OPH (CDS188 and CDS342), and SADH (CDS1771 and CDS3297), providing a genetic basis for the PVA degradation of AC3. Overall, our study reported the first complete genome of *C. nepalense* with PVA-degrading properties, which provided comprehensive insights into the genomic characteristics of this species and increased our understanding of the microbial degradation of PVA.

## MATERIALS AND METHODS

### Isolation and screening of a PVA-degrading bacterium.

Aliquots (1 g) of plastic-attached soil (geographic coordinates 36°20 N, 117°13 E) were diluted serially 10-fold, and 50 μL of the three dilutions (10^−1^, 10^−2^, and 10^−3^) was spread onto LB agar plates (pH 7.2). The petri dishes were incubated at 37°C for 3 days, and the growth of microorganisms was checked every 12 h. Colonies with different morphological features were individually applied to LB agar plates and incubated at 37°C for further growth. This process was repeated three times for purification. The purified bacteria were cultured in LB agar plates, and when the absorbance value at 600 nm was 0.8 to 1.0, the bacteria were inoculated into a screening agar medium using PVA as the sole carbon source, containing PVA (1.00 g/L), (NH_4_)_2_SO_4_ (1.00 g/L), NaCl (0.02 g/L), CaCl_2_ (0.05 g/L), MgSO_4_·7H_2_O (0.10 g/L), FeSO_4_·2H_2_O (0.02 g/L), K_2_HPO_4_ (1.60 g/L), KH_2_PO_4_ (0.20 g/L), and supplemented with agar (15.00 g/L) and bacteria were incubated at pH 7.2 and 37°C for 5 days. The PVA used in this study was purchased from Shanghai Macklin Biochemical Technology Co., Ltd., in China, with a degree of polymerization of 1,700 ± 50 and a degree of alcoholysis of 87.0 to 89.0%.

### Determination of the general characteristics of strain AC3.

The surface morphology of strain AC3 was observed under a Zeiss field emission scanning electron microscope at ×20,000 magnification. To obtain the growth characteristics of strain AC3, estimations were made every 2 h by reading the absorbance value at 600 nm (Synergy HTX multimode reader; Bio Tek). This process was repeated three times.

### Genome sequencing and annotation.

The AC3 genome was sequenced using a PacBio RS II platform and Illumina HiSeq 4000 platform at the Beijing Genomics Institute (BGI, Shenzhen, China). Draft genomic unitigs were assembled using the Celera Assembler against a high-quality corrected circular consensus sequence subread set. To improve the accuracy of the genome sequences, the Genome Analysis Toolkit (GATK) and SOAP tool packages (SOAP2, SOAPsnp, and SOAPindel) were used to make single-base corrections. The estimates for genome completeness and contamination were performed using CheckM (43). Gene prediction and annotation of AC3 and other genomes were performed using a consensus approach based on the RAST server (44).

### Comparative genomic analysis.

The features of the genome and comparisons thereof were performed using BLAST Ring Image Generator (BRIG) (45). The gene families were functionally characterized by COG functional category (46) using eggNOG-mapper software (47). The gene cluster related to secondary metabolism was identified and analyzed using antiSMASH (48) with the default parameters. A GenBank file obtained from the RAST server, containing all the available data, was used as an input to the PathoLogic software from the Pathway Tools version 22.5 with default parameters (49). Syntenic analysis was achieved via ProgressiveMauve (50), which enabled the identification of locally colinear blocks (LCBs) using default parameters. The genes encoding carbohydrate binding and metabolic enzymes were identified using the dbCAN2 database (51) by HMMER search (52).

### Identification of tRNAs, MGEs, and barriers to HGT.

The tRNA loci were collected from the RAST annotation files (44). The prophages were predicted using the online interface of PHAge Search Tool Enhanced Release (PHASTER) (53). The online interface of IslandViewer 4 (54) (integrating three different methods: SIGI-HMM [55], IslandPath-DIMOB [56], and IslandPick [57]) was utilized to identify the genomic islands. Insertion sequences were predicted using the online interface of ISfinder (33). The TA systems were determined by a BLASTp search of the data set from the TADB 2.0 database (58). The RM systems were identified using the online interface of Restriction-ModificationFinder 1.1 (59). The CRISPRs were predicted using the CRISPRCasFinder 4.2.2 software with default parameters (60).

### Identification of potential horizontal genes.

HGTector software was used to identify the potential horizontal genes in the AC3 genome (61). *Chryseobacterium* (rank, genus; taxon ID, 59732) and *Weeksellaceae* (rank, family; taxon ID, 2762318) were set as the self group and the close group, respectively.

### Selection pressure analysis.

MAFFT and ParaAT were used to carry out codon-based alignment of RBH gene pairs between AC3 and *C. takakiae* DSM 26898^T^, as well as AC3 and *C. hispalense* DSM 25574^T^ (62, 63). The nonsynonymous (*Ka*) and synonymous (*Ks*) substitution rates (*Ka*/*Ks*) of these gene pairs were calculated using the YN model in KaKs_Calculator v2.0 (64).

### Biogeographic distribution of *C. nepalense*.

The biogeographic distributions of *C. nepalense*, *C. hispalense*, and *C. takakiae* were then investigated using the IMNGS platform (65), which systematically screens and processes the 500,048 SRA entries (accessed 10 April 2022) for prokaryotic 16S rRNA gene amplicon data sets from diverse niches and builds sample-specific sequence databases. The comparison of the 16S rRNA sequences of *C. nepalense*, *C. hispalense*, and *C. takakiae* with the taxonomically united 16S rRNA database in EzBioCloud (13) revealed that they shared less than 98.4% (closest homolog, *C. takakiae*), 98.82% (closest homolog, Chryseobacterium shandongense), and 99.1% (closest homolog, Chryseobacterium profundimaris) 16S rRNA sequence identities with other related species, respectively. Therefore, the representative 16S rRNA gene sequences of *C. nepalense* (GenBank accession no. KX129820), *C. hispalense* (GenBank accession no. JARQ01000004), and *C. takakiae* (GenBank accession no. KC560016) were queried against all data sets accessible by the IMNGS platform with a minimum DNA size of 200 bp and a threshold of 99%.

### Prediction of potential genes encoding PVA-degrading enzymes.

Annotated protein sequences were aligned with those of known PVA-degrading enzymes described by Wei et al. (22), and protein domain analysis was performed using InterPro 88.0 (66). The functional annotation, sequence BLAST searches, and domain analysis results were integrated to identify PVA-degrading genes.

### Data availability.

The genome data of AC3 were deposited in NCBI GenBank (accession no. CP096203.1).
