# Supplementary material for: Insights into Evolutionary, Genomic, and Biogeographic Characterizations of Chryseobacterium nepalense Represented by a Polyvinyl Alcohol-Degrading Bacterium, AC3
Source: Microbiol Spectr. 2022 Aug 24;10(5):e02179-22. doi: 10.1128/spectrum.02179-22 (PMC9602593; doi:10.1128/spectrum.02179-22)
Supplement: Supplemental file 1 — Fig. S1 to S4. Download spectrum.02179-22-s0001.pdf, PDF file, 0.6 MB [file spectrum.02179-22-s0001.pdf]

SUPPLEMENTAL MATERIAL FIGURE

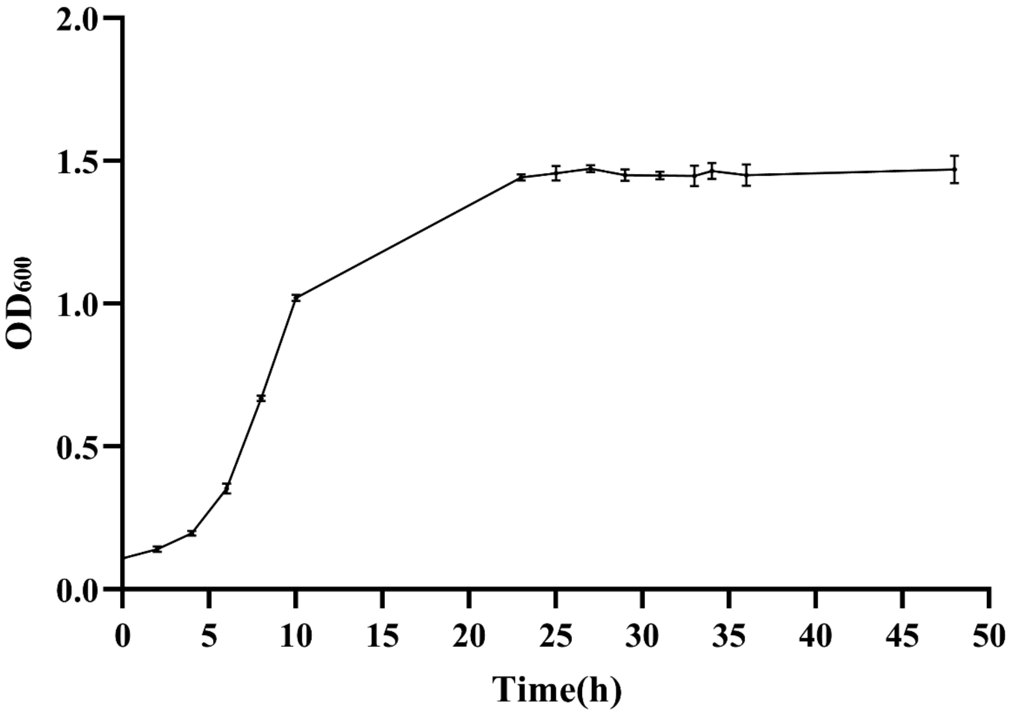

Supplementary Fig. S1. Growth curve of strain AC3 on LB liquid culture medium.

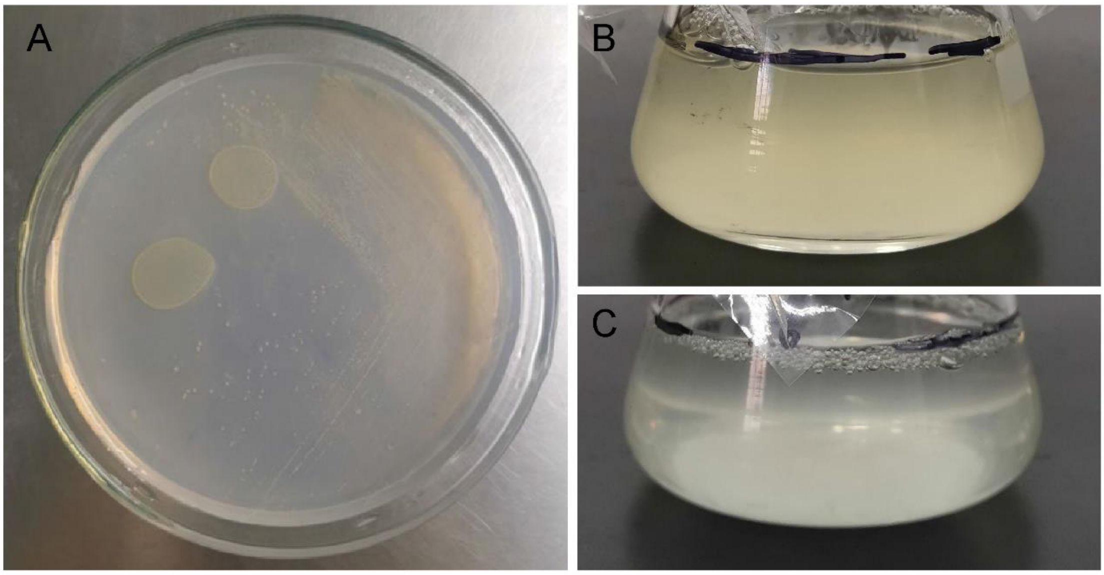

Supplementary Fig. S2. A. The morphological characteristics of the colony on agar medium using PVA as the sole carbon source. When AC3 was grown on a screening agar medium using PVA as the sole carbon source, the colonies became smaller in diameter and lighter in color compared to the colony grown on LB medium. B. Strain AC3 was

grown in a liquid medium with PVA as the sole carbon source. C. Strain AC3 was unable to grow in an inorganic salt liquid medium without PVA as the only carbon source.

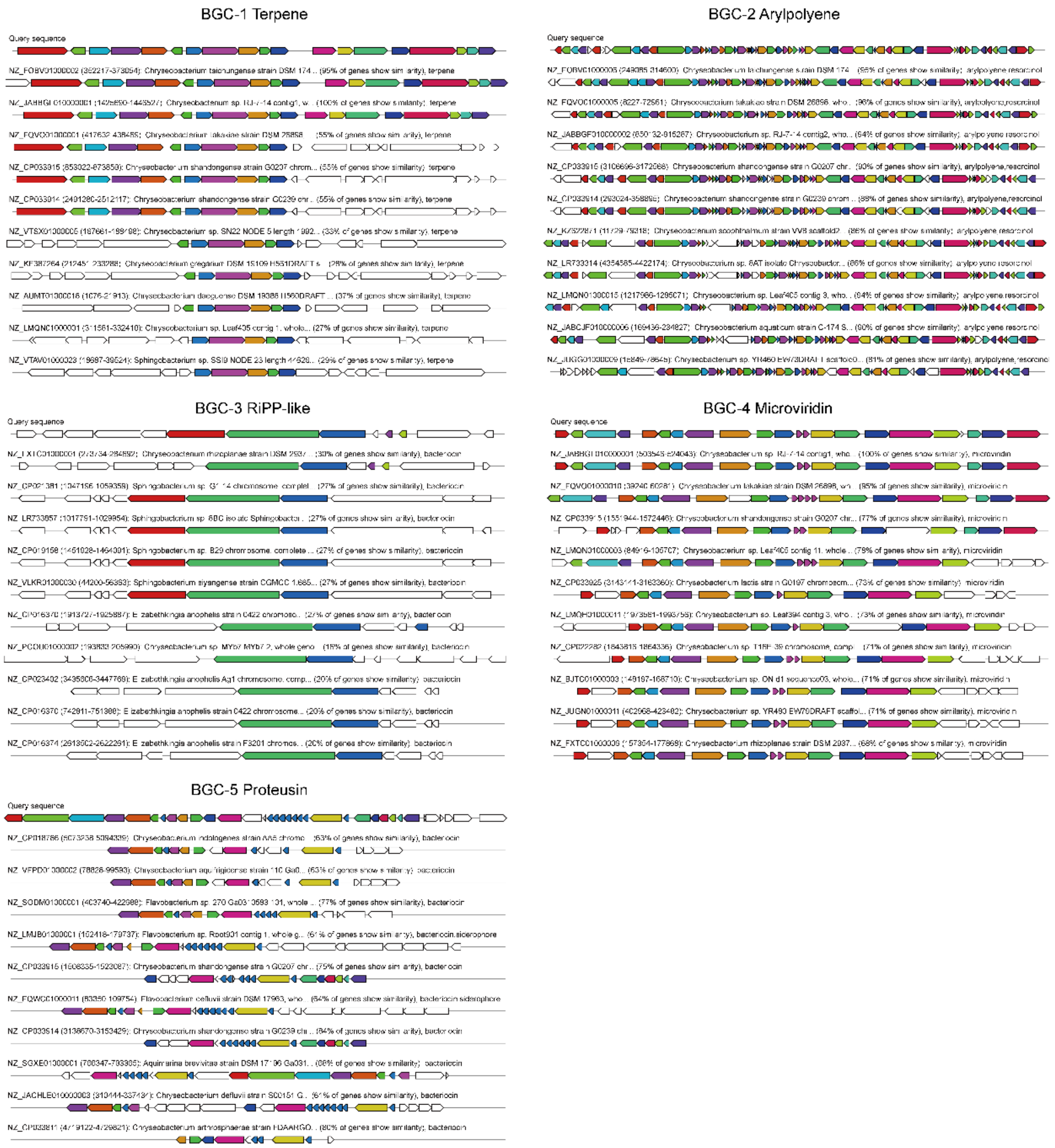

Supplementary Fig. S3. Comparative analysis of biosynthetic gene clusters for secondary metabolism from AC3 and other genomes. Different genes are in different colors, and genes with the same color are homologous to each other.

D-gluconate degradation (GLUCONSUPER-PWY)

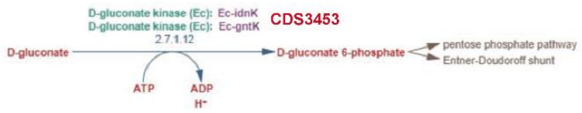

Acyl carrier protein metabolism (PWY-6012)

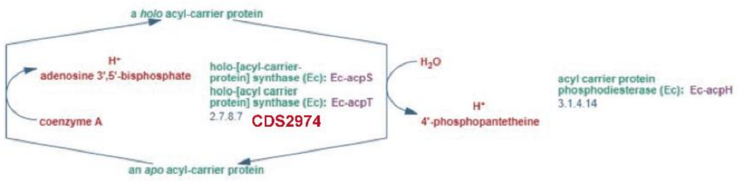

Chitin degradation III (PWY-7822)

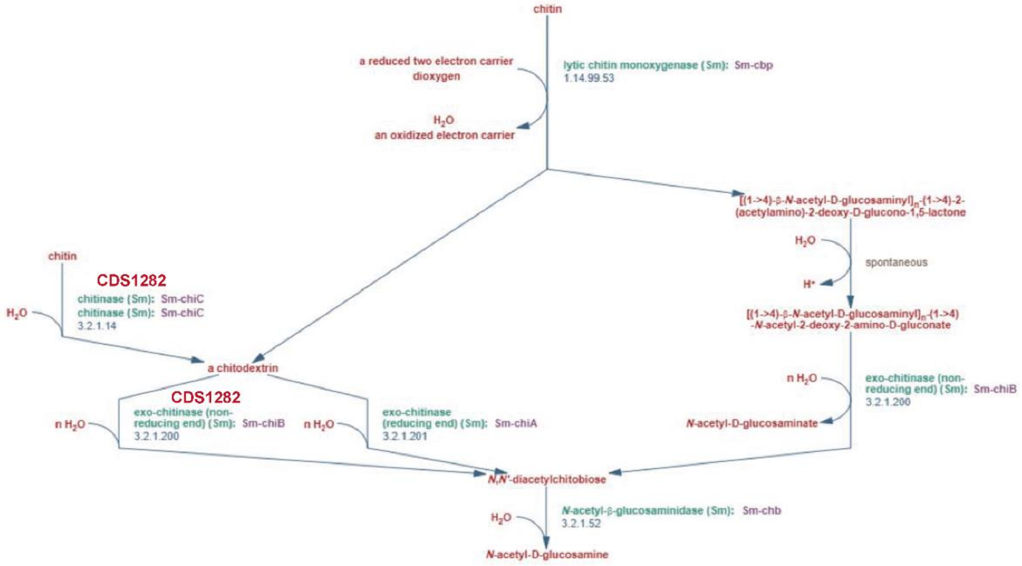

Supplementary Fig. S4. AC3-specific metabolic pathways.
